# Distinct perinatal features of the hyperpolarization-activated non-selective cation current *I*_h_ in the rat cortical plate

**DOI:** 10.1186/1749-8104-7-21

**Published:** 2012-06-13

**Authors:** Arne Battefeld, Nino Rocha, Konstantin Stadler, Anja U Bräuer, Ulf Strauss

**Affiliations:** 1 Institute of Cell Biology and Neurobiology, Center for Anatomy, Charité - Universitätsmedizin Berlin, Charitéplatz 1, 10117, Berlin, Germany; 2 University Rostock, Neurological Clinic, Gehlsheimer Strasse 20, 18147, Rostock, Germany; 3 Netherlands Institute for Neuroscience, Meibergdreef 47, 1105, BA, Amsterdam, The Netherlands

**Keywords:** HCN, Neuronal development, Electrophysiology

## Abstract

**Background:**

During neocortical development, multiple voltage- and ligand-gated ion channels are differentially expressed in neurons thereby shaping their intrinsic electrical properties. One of these voltage-gated ion channels, the hyperpolarization-activated cyclic nucleotide-gated (HCN) channel and its current *I*_h_, is an important regulator of neuronal excitability. Thus far, studies on an early *I*_h_ appearance in rodent neocortex are missing or conflicting. Therefore, we focused our study on perinatal neocortical *I*_h_ and its properties.

**Results:**

In the perinatal rat neocortex we observed a rapid increase in the number of neurons exhibiting *I*_h_. Perinatal *I*_h_ had unique properties: first, a pronounced cAMP sensitivity resulting in a marked shift of the voltage sufficient for half-maximum activation of the current towards depolarized voltages and second, an up to 10 times slower deactivation at physiological membrane potentials when compared to the one at postnatal day 30. The combination of these features was sufficient to suppress membrane resonance in our *in silico* and *in vitro* experiments. Although all four HCN subunits were present on the mRNA level we only detected HCN4, HCN3 and HCN1 on the protein level at P0. HCN1 protein at P0, however, appeared incompletely processed. At P30 glycosilated HCN1 and HCN2 dominated. By *in silico* simulations and heterologous co-expression experiments of a ‘slow’ and a ‘fast’ *I*_h_ conducting HCN channel subunit in HEK293 cells, we mimicked most characteristics of the native current, pointing to a functional combination of subunit homo- or heteromeres.

**Conclusion:**

Taken together, these data indicate a HCN subunit shift initiated in the first 24 hours after birth and implicate a prominent perinatal role of the phylogenetically older HCN3 and/or HCN4 subunits in the developing neocortex.

## Background

In the adult brain the four hyperpolarization-activated cyclic nucleotide-gated (HCN) channel subunits underlying *I*_h_ are differently expressed [1]; in the neocortex HCN1 and HCN2 are predominant. HCN subunits in the brain form both homo- and heteromeric channel tetrameres [2] and differ in their cAMP sensitivity. Whereas the cAMP sensitivity of HCN1 and HCN3 is low, HCN2 and HCN4 are strongly modulated by cAMP [3]. The cAMP sensitivity mainly depends on the cAMP-binding domain that influences voltage-sensitivity, activation times [3] and maximal conductivity [4].

Likewise there is evidence for a considerable contribution of HCN channels in the developing brain. In the hippocampus, an early presence of *I*_h_ facilitates spontaneous oscillations and synchronization between neurons during development around birth [5] and HCN subunits are differentially expressed from postnatal day 1 (P1) onward [6]. In the neocortex, there are several hints for a comparable role of *I*_h_ during development. As for the hippocampus, neocortical synchronization patterns have been described at P0 [7,8]. For instance, non-correlated activity suddenly gets correlated at birth [9] and synchronous plateau assemblies emerge [10]. Such activity may lead to changes in *I*_h_, in particular because HCN expression is correlated between electrically coupled neurons [11] and *I*_h_ is regulated by neuronal activity [12]. Synchronized activity at birth coincides with the existence of structural and functional conditions that render the perinatal cortex prone to excessive excitation [13]. *I*_h_ - if present - may counterbalance excessive excitation, because it powerfully controls the subthreshold somatic efficacy of dendritic excitation in mature layer 5 neurons [14] by acting as a shunt conductance and through voltage-dependent kinetics [15]. In support of such a view, we previously showed that early postnatal environmental alterations influence *I*_h_ in neocortical layer 5 pyramidal neurons of the rat in adulthood [16].

In sharp contrast to its putative role, earliest neocortical data of the *I*_h_-related sag is reported in rats only from P2 onward [17]. In mice, there is some evidence for a prenatal expression of *I*_h_ in very few neurons of the cortical plate followed by a steep increase of HCN-conductance at the end of the first postnatal week [18], but detailed *I*_h_ characteristics are not known for this period. To gain a better understanding of HCN channel function during late prenatal and early postnatal neocortical development, we addressed the properties of *I*_h_ during this period in rat neocortical neurons. In detail, we correlated the developmental changes in current characteristics gathered by whole-cell patch-clamp recordings to the four *I*_h_ underlying subunits. For that, we focused on (nascent) neocortical layer 5 pyramidal neurons, because these neurons show a high density of *I*_h_ in adulthood [19].

Here, we report a robust functional *I*_h_ expression around birth in the rat neocortex. The perinatal *I*_h_ displayed a high cAMP sensitivity and an up to 10 times slower deactivation compared to adult *I*_h_. This was accompanied by a unique expression pattern of HCN subunits. Computer modeling and heterologous expression of HCN channels illustrated that, in principle, a slow and fast HCN subunit can mimic the observed properties around birth. Together these properties prevent resonance at birth, which putatively results in a modulation of neocortical network function.

## Results

### Evidence for a developmentally early cortical *I*_h_ expression in rat

Our initial experiments addressed the inconsistent reports of *I*_h_ presence in the cortical plate around birth. At perinatal stages (E20-P1) we somatically recorded from neurons in the upper and middle cortical plate of which the majority become layer 5 pyramidal neurons [20]. We characterized neuronal membrane parameters (input resistance and resting membrane potential) in artificial cerebrospinal fluid (ACSF). As expected, during development the somatic input resistance (R_in_) decreased and the resting membrane potential (V_m_) dropped to more negative values. Moreover, the estimated cell size by means of the cell capacitance increased (Table 1).

**Table 1:** **Membrane properties measured in ACSF and *I***_**h **_**occurrence in relation to age**

**Age**	**E20**	**P0**	**P1**	**P30**
**V**_**m**_**[mV]**	−58.2 ± 2.4 (n = 16)	−60.5 ± 1.7 (n = 42)	−62.8 ± 1.2 (n = 13)	−68.4 ± 0.9 (n = 25)
**R**_**in**_**[MΩ]**	2756 ± 357.4 (n = 16)	2204 ± 254.6 (n = 42)	895.0 ± 45.0 (n = 13)	99.2 ± 6.5 (n = 25)
**C [pF]**	35.3 ± 3.6 (n = 16)	44.4 ± 1.7 (n = 50)	66.8 ± 2.9 (n = 17)	167.3 ± 9.4 (n = 35)
***I***_**h**_**presence**	11 out of 23 (48%)	80 out of 106 (75%)	29 out of 31 (94%)	All, n = 39 (100%)

Resting membrane potential (V_m_) and input resistance (R_in_) were recorded during control measurements in ACSF before pharmacological isolation of *I*_h_. The presence of the hyperpolarization-activated current (*I*_h_) was assumed for neurons showing either a voltage sag or inward rectifying current upon membrane hyperpolarization.

**Figure 1:**
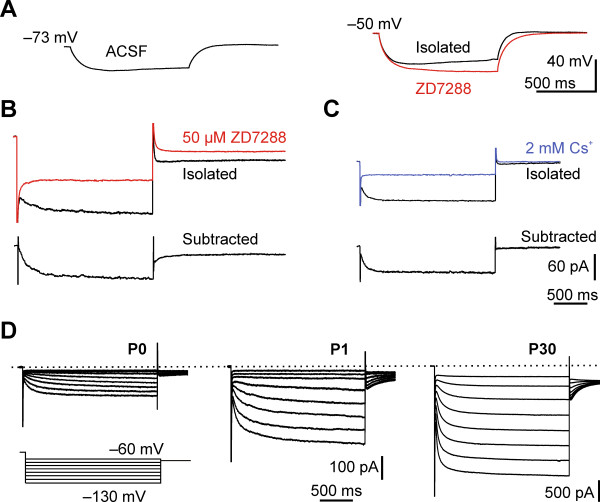
**Presence of*I***_**h**_**in cortical plate neurons at P0 and its developmental increase. (A)***Left*: A distinct sag was observed at P0 after current injection in ACSF. *Right*: Pharmacological isolation of *I*_h_ depolarized the membrane and the sag was still present. Application of ZD7288 abolished the sag (*red trace*). Membrane voltages were recorded in the presence of 100 μM intracellular cAMP. (B) and (C) Reduction of the inward current at P0 by *I*_h_ blockers. **(B)***Top*: Pharmacological isolated *I*_h_ traces of a P0 neuron before (*black*) and 450 seconds after bath application of 50 μM ZD7288 (*red*). *Bottom*: ZD7288 sensitive current component obtained by subtracting the remaining current after ZD7288 application from the initial hyperpolarization induced current. **(C)***Top*: Current after pharmacological isolation of *I*_h_ (*black trace*) and after bath application of 2 mM Cs^+^ for 360 seconds (*blue trace*). *Bottom:* Cs^+^ sensitive current obtained by the same method as described for (B). Currents were activated by stepping to a command voltage of −120 mV (B) or −130 mV (C) from a holding potential of −40 mV. Note that tail currents were also abolished by the respective blockers. Scale bars in (C) also apply to (B). Displayed currents were recorded in the intracellular presence of 100 μM cAMP. **(D)** Families of pharmacologically isolated and non-post hoc processed *I*_h_ (see Methods) in the absence of cAMP in neocortical neurons at developmental stages P0, P1 and P30 in response to 2-second voltage steps from −60 to −130 mV in 10 mV intervals, elicited from a holding potential of −40 mV (*bottom*). Note that traces depicted from P0 and P1 neurons are magnified approximately eightfold for clarity (see scale bars). Tail currents are cut at 500 ms, for a detailed analysis see Figures 2 and 4. The indicated time scale applies for all currents in (D).

**Figure 2:**
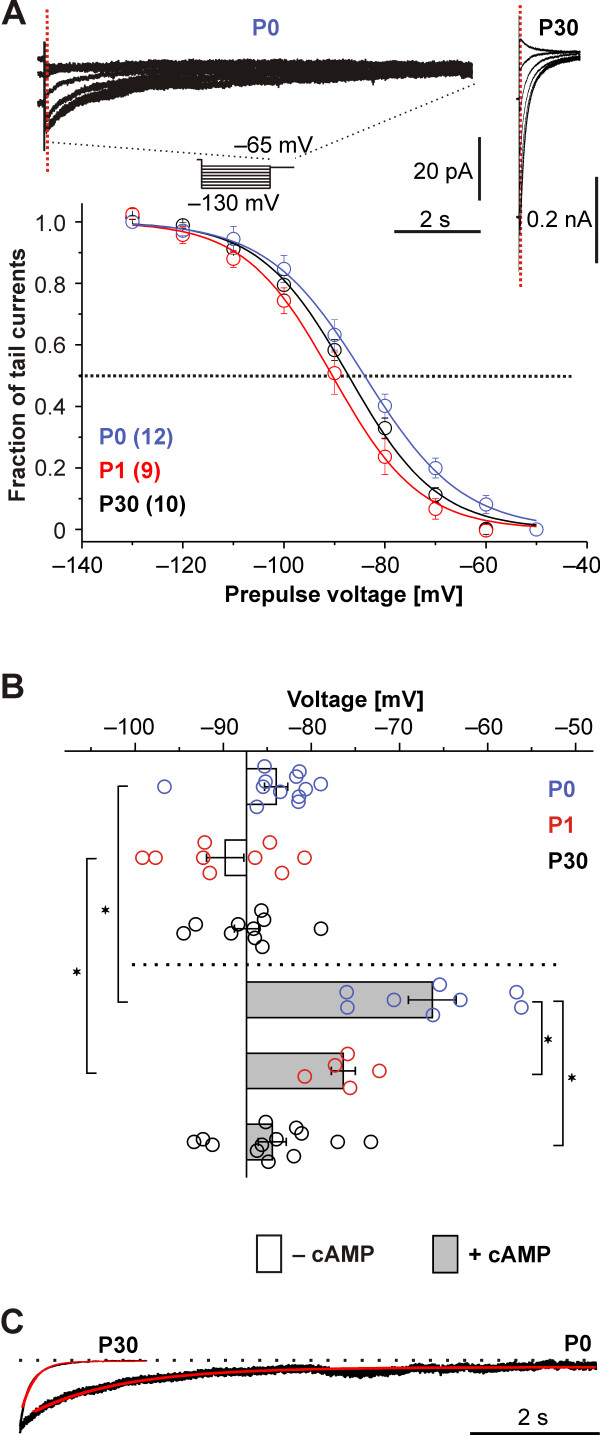
***I***_**h**_**voltage dependence and its modulation by cAMP. (A)***Top*: Family of tail currents from neocortical neurons at P0 (*left*) and at P30 (*right*). For both examples, different durations of the tail currents are displayed on return to −65 mV from preceding 2-second voltage steps between −130 and −60 mV (see voltage protocol). Tail current amplitudes were measured after the cessation of capacitive transients (*dotted vertical line*). The time scale is representative for both ages. *Bottom*: Population data of steady-state activation curves without intracellular cAMP, depicted as mean relative tail currents, plotted against the preceding test potential. For illustration purposes the means were fitted using a Boltzmann function, although during analyses each single neuron was fitted. For individual experimental values compare (B). **(B)** Population data on the modulation of the half-maximal activation voltage (V_1/2_) by cAMP at P0, P1 and P30. The V_1/2_ did not differ between ages when cAMP was absent from the intracellular solution (*open bars*). Adding 100 μM cAMP intracellularly shifted the V_1/2_ at P0 and P1 to more depolarized voltages, but did not change the V_1/2_ at P30 (*lower part, grey bars*). **(C)** Age dependence of the current deactivation at −120 mV for a P0 and a P30 neuron in a scaled example. The current was fitted by a single exponential equation (*red*) and showed a prolonged deactivation at P0. *Indicates significance *P* < 0.05 (ANOVA with Tukey post-hoc test).

We expected small current amplitudes, thus for initial recordings we included 100 μM cAMP in the pipette to improve voltage independent maximum open probability of *I*_h_[21]. At P0 we detected a small, but visible sag in ACSF that was more pronounced after elevating [K^+^_o_ to 10 mM and pharmacologically blocking a number of intrinsic (Na_V_, Ca_V_, K_V_, K_IR_) and synaptic (AMPA, NMDA, GABA_A_) currents (isolated, Figure 1A). After application of 50 μM ZD7288 the sag disappeared, further suggesting that it was caused by *I*_h_ (Figure 1A). For subsequent analyses we recorded the current in voltage clamp. At first, we aimed to assure the identity of the current. For this purpose, we bath-applied either 50 μM ZD7288 (Figure 1B) or 2 mM cesium (Figure 1C) after pharmacological isolation. Both blockers markedly reduced the slow-activating inward-rectifying current in all neurons tested across ages (ZD7288, n = 11; 2 mM Cs^+^, n = 24) as shown for *I*_h_[22]. The number of neurons in which we observed a sag or an inward-rectifying current increased in the first two days after birth to almost 100% (Table 1).

For further characterization, we pharmacologically isolated *I*_h_ without intracellular cAMP to reduce experimentally induced alterations (henceforth Figure 1D unless otherwise stated). The isolated *I*_h_ increased in magnitude (Figure 1D): amplitudes and densities (current amplitude normalized to capacitance) of *I*_h_ were small in perinatal neurons, compared to the ones at P30. *I*_h_ amplitude estimated at −120 mV increased significantly from −61.4 ± 9.7 pA at P0 (n = 12), –160.9 ± 21.5 pA at P1 (n = 8) to −948.2 ± 182.9 pA at P30 (n = 10) (ANOVA, *P* < 0.001). The *I*_h_ density increased from 1.3 ± 0.2 pA/pF at P0 (n = 12) and 2.2 ± 0.3 pA/pF at P1 (n = 8) to 4.5 ± 0.6 pA/pF at P30 (n = 10), illustrating that this increase in *I*_h_ amplitude was not merely related to an increase in cell size. The reversal potential as a rough measure of ion selectivity was comparable at P0 (V_rev_ = −40.0 ± 3.2 mV) and P30 (V_rev_ = −37.3 ± 4.2 mV, Mann–Whitney U, *P* = 0.14, estimated in a set of experiments with 100 μM cAMP). Taken together, these data point to an early, even prenatal, functional expression of a slow depolarizing current in the cortical plate of rats, which we identified as *I*_h_ and which increased rapidly after birth.

### Unique properties of *I*_h_ in perinatal neurons are partially due to differences in cAMP sensitivity

We clearly identified *I*_h_, however, the current displayed two characteristics with a strict perinatal prominence uncommon for adult cortical *I*_h_. First, the perinatal voltage dependence of *I*_h_ activation was strongly dependent on cAMP, the canonical HCN modulator. Without intracellular cAMP we calculated half-maximum activation voltages (V_1/2_) that were similar at the measured time points of P0, P1 and P30 (V_1/2_: at P0 −84.0 ± 1.3 mV, k = 9.6 ± 1.1, n = 12; at P1 −89.8 ± 2.1 mV, k = 7.3 ± 0.6, n = 9; at P30 −87.4 ± 1.4 mV, k = 8.6 ± 0.4, n = 10; Figure 2A, for intergroup comparisons see Figure 2B). However, the inclusion of intracellular cAMP (100 μM) markedly depolarized the half-maximum steady state voltage activation V_1/2_ at P0 (−66.3 ± 2.7 mV, k = 8.1 ± 0.7, n = 8) but less at P1 (−76.4 ± 1.4 mV, k = 11.2 ± 1, n = 5; Figure 2B). At P30 cAMP marginally shifted the V_1/2_ to −84.4 ± 1.6 mV, k = 11.7 ± 0.8, n = 13 (ANOVA for all conditions as indicated in Figure 2B: V_1/2_: *P* < 0.0001; k: *P* = 0.002). Our results at P30 are in line with previous work that showed that at mature stages (P28 to P70) the cAMP sensitivity disappeared in neocortical neurons in layer 5 [23].

Second, *I*_h_ deactivation observed after stepping back to physiological membrane potentials from a fully activating 2-second prepulse of −130 mV or −120 mV, which followed a mono-exponential time course after an initial delay phase, was about 10 times slower at P0 (*τ*_deact_ = 1238 ± 83 ms, n = 12) compared to P30 (*τ*_deact_ = 126 ± 24 ms, n = 10; Figure 2C). At both ages, the open channels conducting the tail currents are very likely in a locked-open mode, because the previous hyperpolarizations should be long and strong enough [24].

**Figure 3:**
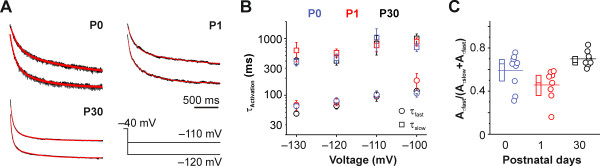
**Activation kinetics of pharmacologically isolated*I***_**h**_**slightly accelerated with age. (A)** Representative *I*_h_ activation traces of P0, P1 and P30 neurons in response to hyperpolarizing step potentials of −110 mV and −120 mV *(voltage protocol: bottom right*). The traces are depicted from approximately 20 milliseconds after step onset (skipping the capacitive relaxation) to the end of the 2-second step. Red lines are double exponential fits. Note that current amplitudes have been normalized for comparability. **(B)** Activation time constants speeded up only marginally from P0 to P30, when measured somatically. Displayed are fast (*circles*) and slow (*squares*) time constants of a double exponential fit. Ages in (B) and (C) are coded in colors: blue - P0, red - P1, black - P30. **(C)** Population data and 25 to 75% box plots on the amplitude contribution of fast-activating *I*_h_ components to the whole current (n = 8 - 9) for the studied time points. Solid lines through boxes and data represent means.

However, in the same somatic recordings the opening kinetics did not accelerate in a similar manner during development (Figure 3A). *I*_h_ activation was best fitted by a double-exponential equation except for two neurons at P0, which were excluded from the analyses. In the remaining neurons, the fast activation time constant (*τ*_fast_) merely showed a tendency towards acceleration from 79.8 ± 10.5 ms at P0, n = 10 to 64.8 ± 7.8 ms, n = 10 at P30 (ANOVA, *P* = 0.46) at full *I*_h_ activation (command potential of −120 mV). In a similar fashion, the slow activation time constant (*τ*_slow_) only marginally accelerated from 450.8 ± 84.7 ms at P0 (n = 10), to 404.6 ± 65.7 ms at P30 (n = 10; ANOVA, *P* = 0.29, Figure 3B). This is less than reported for hippocampal CA1 neurons between P1 and P20 [6], but our somatic recordings might underestimate the acceleration of *I*_h_ at P30 because of poor voltage clamp control at sites more distal to the soma [25]. In fact, direct dendritic recordings at adult stages revealed much faster activations (*τ*_fast_ ~ 20 ms; [23]). To roughly estimate the contribution of the fast activation components to the current in individual cells, we calculated the amplitude fraction of *τ*_fast_ (A_*τ*fast_/(A_*τ*fast_ + A_*τ*slow_), Figure 3C), which were 0.6 ± 0.04 (n = 10) at P0, 0.5 ± 0.05 (n = 8) at P1, and 0.7 ± 0.03 (n = 8) at P30 (without intracellular cAMP), indicating a similar contribution of fast and slow component to the somatically recorded *I*_h_.

In conclusion, perinatal *I*_h_ shows two novel and coinciding characteristics, a pronounced cAMP dependence of the voltage dependence of activation and a distinct slowing of current deactivation.

### Neither cAMP- nor PLC-dependent mechanisms account for the slowing in *I*_h_ deactivation

**Figure 4:**
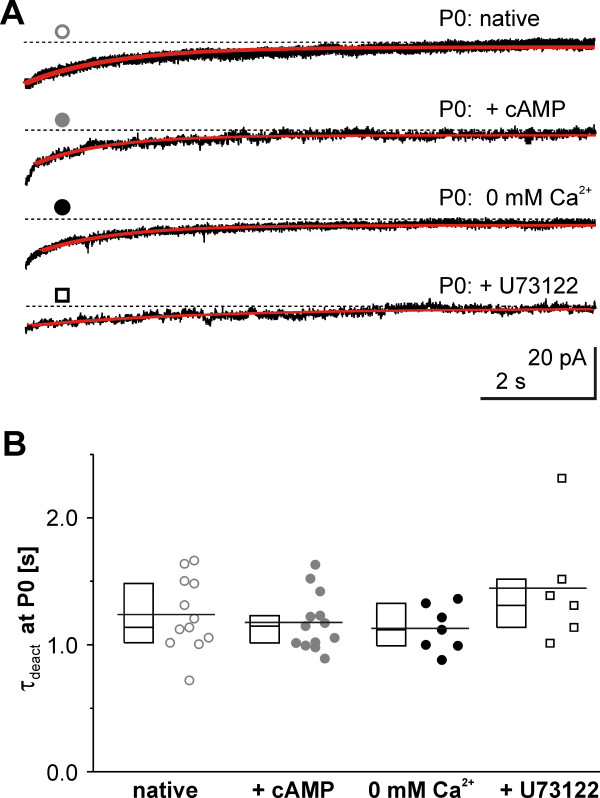
**Robust*I***_**h**_**deactivation kinetics at P0. (A)** Examples of *I*_h_ deactivations at −65 mV from a preceding activation step of −130 mV and corresponding single exponential fits at P0 under varying conditions. The course of *I*_h_ deactivation did not change when intracellular modulators (**+**cAMP or + U73122) were included or extracellular Ca^2+^ omitted (0 Ca^2+^). Symbols correspond to the coding in (B). **(B)** Deactivation time constants were not changed after experimental alterations of intracellular cAMP (100 μM, *grey*-*filled circles*), omitting extracellular Ca^2+^ (*black*-*filled circles*), or by adding the intracellular PLC blocker U73122 (*open squares*) compared to native P0 conditions (*open grey circles*). Data are presented with 25 to 75% box plots (left of respective data). The mean for each condition is indicated by a line comprising the box and individual data points.

Cyclic AMP binding to HCN channels thermodynamically shifts the V_1/2_ by relief of autoinhibition and additionally influences kinetic properties. The deactivation is influenced by trapping the channels in an open state position therewith prolonging the kinetics [26]. In our experiments at P0, however, supramaximal intracellular cAMP concentration did not further influence the speed of *I*_h_ deactivation (*τ*_deact_ = 1176 ± 62.4 ms, n = 13, ANOVA, *P* = 0.21, Figure 4A, B) after full current activation. Additionally, including cAMP at P0 did not influence the speed of current activation (*τ*_fast_ = 68.5 ± 7 ms, *τ*_slow_ = 458.1 ± 97.2 ms, n = 6, *P* < 0.1, when compared to the values obtained w/o intracellular cAMP) despite the prominent shift of V_1/2_. This disparity would be in line with the suggestion of two different mechanisms of cAMP action on HCN channels [26].

Another canonical *I*_h_ modulator, phosphatidylinositol 4,5-biphosphonate (PIP_2_), could change the time course of deactivation. Therefore, we attempted to interfere with this phosphoinosid by phospholipase C (PLC) blockade, known to either hydrolyze PIP_2_ or activate downstream signaling pathways that increase PIP_2_[27]. However, inhibiting PLC by U73122 did not change deactivation velocity (*τ*_deact_ = 1446 ± 188 ms, n = 6, ANOVA, *P* = 0.21, Figure 4A, B). In line with published data [27] we observed a hyperpolarization of voltage sensitivity induced by U73122 at P0 (V_1/2_ = −90.6 ± 1.8 mV, k = 8.5 ± 0.6, n = 4), indicating a role of PIP_2_ in the control of *I*_h_ voltage sensitivity at P0.

Next, we considered a Ca^2+^ influx through open HCN channels [28] or non-blocked Ca^2+^ channels with a subsequent intracellular Ca^2+^ transient (which might circumvent the rather slow Ca^2+^-chelator ethylene glycol tetraacetic acid (EGTA) in our intracellular solution). To investigate the putatively HCN influencing role of consecutive Ca^2+^ triggered processes we modified the Ca^2+^ concentration in the bath solution. This revealed that the deactivation was not sensitive to Ca^2+^ changes in the physiological range, as the deactivation time did not differ regardless of the extracellular Ca^2+^ concentration (for 0 mM: *τ*_deact_ = 1128 ± 68.6 ms, n = 7, for 2.5 mM: *τ*_deact_ = 1025 ± 45.6 ms, n = 5 and 5 mM: *τ*_deact_ = 1312 ± 301 ms, n = 2, for 2.5 and 5 mM pooled: *τ*_deact_ = 1107 ± 90 ms, Figure 4A, B).

In summary, although cAMP and PLC blockade shifted the voltage sensitivity of *I*_h_ activation, none of the above modulators influenced the *I*_h_ deactivation kinetics.

### Perinatal HCN subunit expression

Based on our foregoing results, we assumed structural differences, that is distinct subunit arrangements, at perinatal stages for several reasons. First**,***I*_h_ characteristics such as kinetics, voltage- and cAMP- sensitivity depend on HCN subunit composition. Second, HCN subunit expression is at least transcriptionally controlled [29]. And third, none of the probed conventional modulators entirely explained the characteristics at P0.

**Figure 5:**
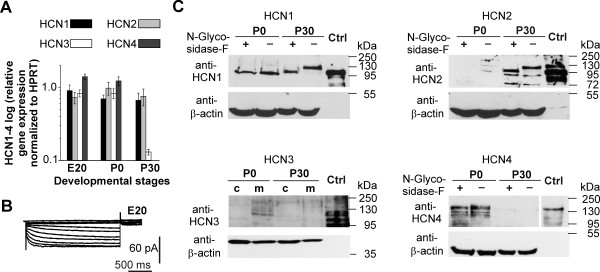
**Developmental expression change of HCN subunits. (A)** Quantitative real-time PCR of rat neocortical tissue for developmental stages E20, P0 and P30 revealed age-dependent changes in HCN3 and HCN4 mRNA expression levels, whereas the expression of *HCN1* and *HCN2* remained stable. Data were normalized to respective HPRT expression. Experiments were repeated three times and error bars represent SEM. **(B)** A family of pharmacologically isolated *I*_h_ at E20 recorded from a holding voltage of −40 mV and activation potentials between −40 and −130 mV confirmed a functional expression of *I*_h_ already at E20. **(C)** Western blot analysis from neocortical lysates for HCN1-HCN4 proteins at P0 and P30. For all blots we used anti-beta-actin as loading control and the corresponding subunits (HCN1-HCN4) over-expressed in HEK293 cells as positive controls (ctrl, *right lanes*). HCN1, HCN2 and HCN4 membrane protein fraction probes were treated with peptide:N-glycosidase F (PNGase F) in order to remove N-linked glyocsylation. HCN1 was detected in both, P0 and P30 protein extracts, but at higher molecular weights at P30. Deglycosylation at P30 shifted the band to lower molecular weights. However, no shift was observed at P0. Note that the bands at P0 still do not match the bands of deglyocsylated P30 protein. HCN2 was not detectable at P0 but at P30. Deglyocosylation of protein extracts shifted the top band to a lower molecular weight in the P30 samples. HCN4 was only detectable at P0 and the upper band was partially reduced by PNGase F treatment. HCN3 was present in membrane extracts of P0 neocortex tissue whereas no protein was detectable in P30 membrane fractions.

Initially, we analyzed the HCN subunit expression pattern by quantitative real-time PCR (qRT-PCR). In the perinatal rat cortex the expression levels markedly differed from the more mature cortex (Figure 5A). Particularly HCN3 and HCN4 mRNAs were strongly expressed perinatally but dramatically decreased at P30. In the same time, HCN1 and HCN2 mRNA levels did not change (Figure 5A). Notably, the prenatal mRNA expression was accompanied by a small but measurable *I*_h_ (at E20) under isolating conditions (Table 1, Figure 5B).

Considering that mRNA levels do not necessarily reflect protein levels for HCN subunits [29], we subsequently set out to narrow down the subunits putatively involved by western blot analysis from neocortical membrane protein lysates at P0 and P30 (Figure 5C). HCN protein expression was age-dependent and differed substantially from mRNA levels. The protein expression of both ‘slow’ subunits HCN3 and HCN4 grossly followed their mRNA levels, but HCN1 and HCN2 did not. In detail, we detected immunoreactive bands for HCN1, HCN3 and HCN4 at P0, but solely HCN1 and HCN2 at P30, indicating that HCN2 does not contribute to *I*_h_ at P0. However, HCN3 protein expression was only present at very low levels in membrane fractions of rat neocortical protein lysates at P0. HCN1 was the only subunit present in the membrane fraction at P0 and P30, though at P0 its immunoreactive band appeared at a lower molecular weight (approximately 100 kDa), in comparison to P30 (approximately 125 kDa). This was caused by a lack of N-linked glycosylation at P0, as treatment with PNGase F shifted the band at P30 to a lower molecular weight, but not at P0. Notably, both HCN1 bands at P0 showed a lower molecular weight compared to the deglycosylated band at P30. Given the conserved consensus site for N-glycosylation in the extracellular loop between S5 and the pore helix of all four HCN channels [30] we also checked HCN2 and HCN4. The very low HCN3 protein expression in neocortical tissue precluded N-glycosylation analyses. For HCN2, multiple immunoreactive bands were detectable at P30, as in the HCN2 over-expressing HEK293 cell protein lysate used as positive control. After PNGase F treatment of P30 protein lysates the upper band shifted to a lower molecular weight and the immunoreactivity of the additional lower bands increased. For HCN4 membrane fractions of P0 cortical plate showed two bands higher than 130 kDa and after deglycosylation, the upper band was abolished.

In summary, we observed developmental changes in HCN subunit mRNA and protein expression with alterations in N-linked glycosylation of HCN1.

### HCN subunit rearrangements are sufficient to cause all perinatal features

**Figure 6:**
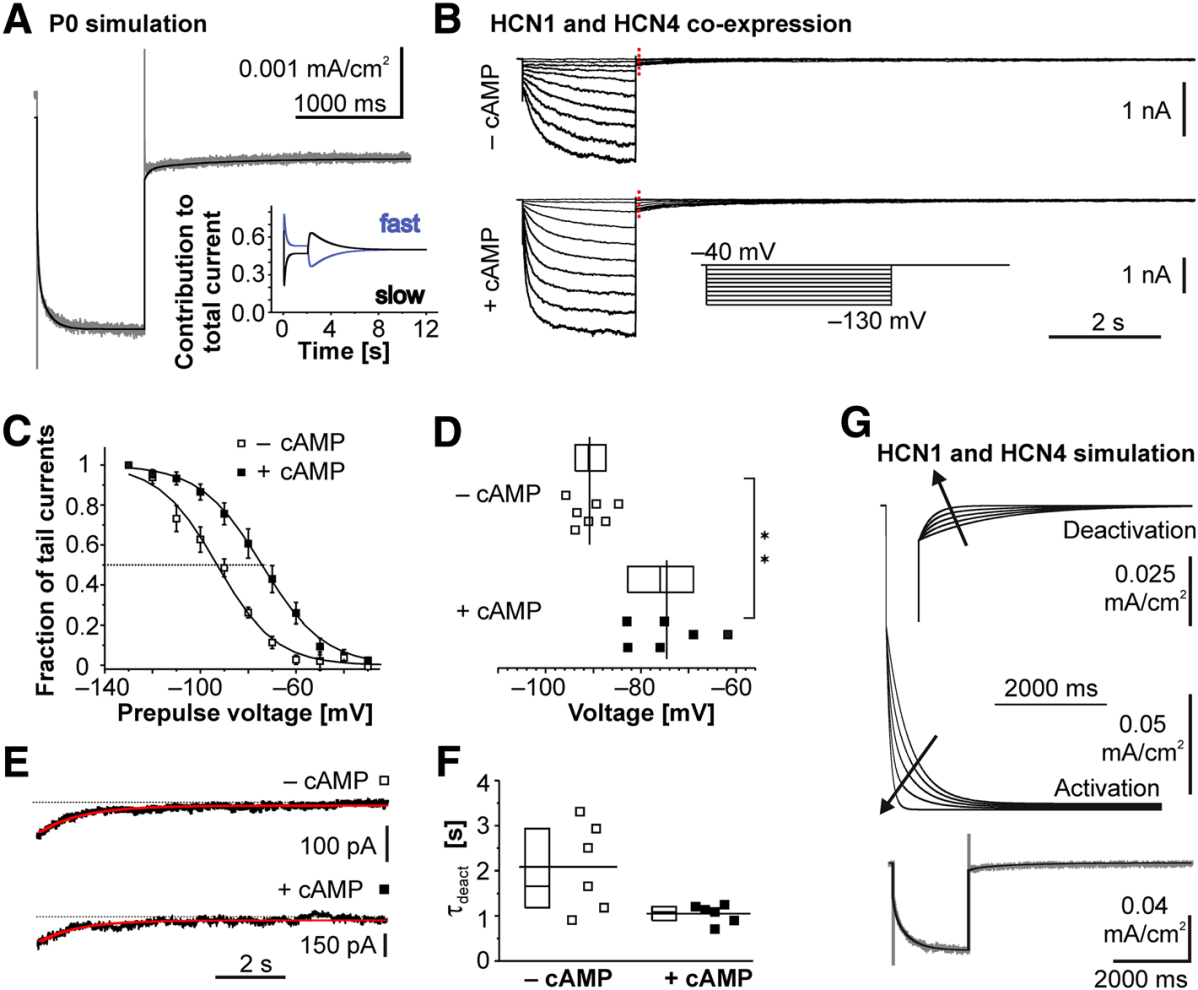
**Simulation of*I***_**h**_**properties and co-expression of HCN1 and HCN4 subunits in HEK293 cells. (A)** The simulated combination of a fast- and a slow-activating HCN subunit replicates the characteristic kinetic properties (fast activation and slow deactivation) at P0. Simulation of *I*_h_ conducted by a fast- and slow-activating HCN subunit (*black trace*) elicited by a −130 mV step for 2 seconds (*bottom*) superimposed onto a trace obtained by the same voltage protocol in a neocortical neuron at P0 (*grey trace*). *Inset:* the kinetic behavior is explained by the time-dependent contribution of a fast (*blue line*) and a slow (*black line*) component. **(B)** Families of HCN1- and HCN4-mediated *I*_h_ recorded from HEK293 cells co-expressing these subunits. Currents in the absence (*top*) and presence (*bottom*) of cAMP were activated from a holding potential of −40 mV to command potentials between −40 and −130 mV of 2 seconds length (*inset*). Tail currents were recorded after stepping back to −40 mV and showed a slow deactivation and tail current amplitudes were measured at the time point indicated by the dotted red line. **(C)** Voltage sensitivity of HCN1 and HCN4 subunits co-expressed in HEK293 cells. Activation curves in the presence (*closed box*) and absence (open box) of cAMP are depicted as mean ± SEM. For illustration purposes the population means were fitted using a Boltzmann function. For evaluation of the voltage sensitivity individual neurons were considered (see D). Intracellular cAMP clearly shifted the half-maximal activation voltage by approximately 16 mV. **(D)** Population data on half-maximal activation voltage V_1/2_ for the HCN1 and HCN4 mediated *I*_h_ showed the marked difference (*P* <0.01) between experimental conditions without (*top*) and with (*bottom*) 100 μM intracellular cAMP. **(E)** Representative current deactivation of HCN1 and HCN4 co-expressing HEK cells (*black*) without (*top*) and with 100 μM cAMP (*bottom*) displayed with an overlay of a single exponential fit in red. **(F)** Population data on *I*_h_ deactivation kinetics in HCN1- and HCN4-expressing HEK293 cells without (*left*) and with (*right*) 100 μM intracellular cAMP. **(G)** Subunit ratio determines *I*_h_ kinetics. Simulation of different ratios of fast and slow HCN subunits with two independent first-order kinetic models. Displayed fractions of the fast subunit were (inside to outside - *arrow direction*) 0, 0.30, 0.60, 0.90 and 1.00, respectively. *Bottom*: The simulation (*black trace*) was experimentally supported by a co-expression of HCN1 and HCN4 (*grey trace*) in HEK293 cells as in (B) that showed similar results.

In order to study the possibility of an involvement of one of the very slow deactivating channel subunits, either HCN3 or HCN4, in the conduction of *I*_h_ at P0, we simulated a current through a combination of a fast and a slow HCN subunit by combining two independent first-order kinetic model components. We based the simulation of the fast component, *I*_hfast_, on our previous rHCN1 data [31]. The slow component, *I*_hslow_, was subsequently used to adjust *I*_htot_ to the *I*_h_ measured in the rat P0 neurons. This resulted in a reasonable fit (Figure 6A) when the proportion of the fast HCN component on the whole HCN conductance was 0.53. Nevertheless, the proportion of *I*_hfast_ on *I*_htot_ substantially varied over time because of the differences in channel kinetics, thus explaining the fast activation and slow deactivation observed on the simulated trace (Figure 6A, inset). Because our kinetic model could be easily fitted to the experimental data in P0 neurons, we hypothesized that characteristic channel activation and deactivation kinetics are due to fast-activating (dominating the activation phase of *I*_h_) combined with slow-deactivating HCN channels (dominating the deactivation phase of *I*_h_).

As proof of principle, we tried to reproduce the *I*_h_ characteristics at P0 by co-expressing a fast and a slow HCN subunit in HEK293 cells. As possible components we chose HCN1, because it is the fastest activating subunit, and HCN4, to account for the slow deactivation and strong cAMP sensitivity at P0, as well as its reduced expression at later stages. The resulting *I*_h_ resembled the one in perinatal rat neurons for the most parts (Figure 6B-F). In detail, with supra-maximal cAMP intracellularly, *I*_h_ activated fast (at −130 mV: *τ*_fast_ = 49 ± 6 ms, *τ*_slow_ = 288 ± 35 ms) and deactivated slow (*τ*_deact_ = 1.1 ± 0.1 seconds, n = 6, respectively) and omitting cAMP hyperpolarized the half maximal activation voltage (from V_1/2_ = −74.6 ± 3.3 mV, n = 6 to V_1/2_ = −90.8 ± 1.5 mV, n = 7, Mann–Whitney *U* test *P* < 0.01, Figure 6C, D) without changing the slope (k = 12.0 ± 0.6, n = 6 vs. k = 12.6 ± 1.8, n = 7, Mann–Whitney *U* test *P* = 0.7). However, in contrast to the situation at P0, omitting cAMP also slowed the current activation (at −130 mV: *τ*_fast_ = 110 ± 22 ms, *P* < 0.01, *τ*_slow_ = 913 ± 106 ms, *P* < 0.01 - both Mann–Whitney *U* test), but not the deactivation (*τ* = 2.1 ± 0.4 seconds, Mann–Whitney *U* test *P* = 0.07). We simulated various contributions of HCN1 to *I*_h_ for the case of HCN1 and HCN4 co-expressing HEK293 cells at −130 mV in analogy to our modeling approach in P0 neurons. The contribution of the HCN1 subunit clearly determined the current kinetics (Figure 6G). Meeting the kinetics of an example trace from a HCN1- and HCN4-expressing HEK cell required a HCN1 fraction of 0.24 (Figure 6G bottom).

In summary, our *in silico* simulations and heterologous over-expression experiments demonstrate, that arrangements of a fast and slow HCN channel subunit suffice to explain the unique perinatal features of *I*_h_.

### Functional consequences

**Figure 7:**
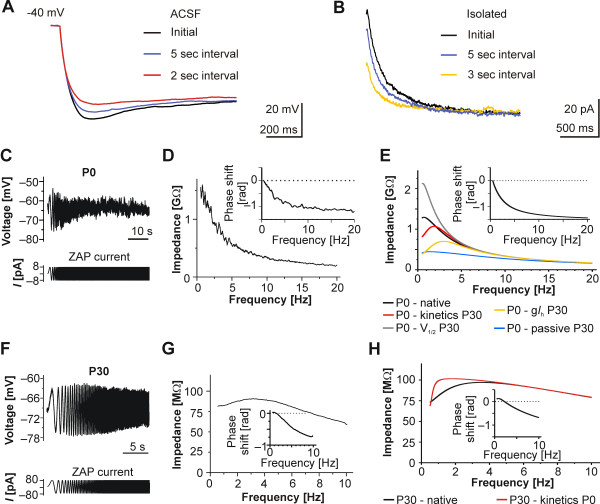
**Functional implications and resonance behavior of layer V neocortical neurons at P0 and P30. (A)** Varying the interpulse interval in the current-clamp between ‘infinite’ (initial), 2 seconds or 5 seconds reduced the sag amplitude as a result of the slowed deactivation. Depicted traces were recorded after injection of −50 pA. **(B)** Dependence of *I*_h_ activation and an instantaneous component on the interval of consecutive voltage steps to −130 mV of an ‘infinite’ (initial), 3-second or 5-second interval. **(C/F)** Examples of the membrane voltage of a neuron at P0 (C, *top*) and at P30 (F, *top*) as a response to ZAP current injection (*bottom*). Note the lack of a distinguishable peak despite the frequency increase to 20 Hz in P0 neurons. **(D/G)** Calculated impedance in relation to the frequency for the cells depicted in (C) and (F). Note the absence of a distinct membrane resonance peak and the relatively high impedance values at P0 (D) as compared to the distinct peak at approximately 3 Hz and lower impedance at P30 (G). At both age groups data points were averaged from three consecutive ZAP recordings. *Insets:* Impedance phase shifts in relation to the frequency. **(E/H)** Modeling (membrane voltage of −70 mV) using the entire parameter set for either a P0 or P30 neuron (*black lines*) mimicked the results of the ZAP recordings of rat neocortical neurons in brain slices. (E) Changing the kinetics to values of a P30 neuron (*red line*) or adjusting the conductance to a P30 neuron (*yellow line*) resulted in distinct resonance peaks, whereas replacement of the half-maximal activation by P30 values (*grey line*) or substituting the passive properties to values at P30 (*blue line*) led to massive impedance changes. (H) In contrast, replacing the simulation values at P30 by only the kinetic properties of a P0 neuron still resulted in a resonance peak, though less distinct (*red line*). *Insets:* Impedance phase shifts in relation to the frequency calculated from the simulated data. Only neurons at P30 showed a peak positive phase value and therewith a crossover from positive to negative phase in the impedance phase profiles.

As long deactivation times might influence responses on repetitive stimulations, we shortened the hyperpolarization intervals to less than 30 seconds. This led to an interval-dependent reduction of the sag (Figure 7A). Consecutive voltage clamp recordings revealed that this was due to an increase in the amount of an instantaneous current component at shorter interpulse intervals while the steady-state current remained unchanged (Figure 7B). We attribute the instantaneous current component to still open HCN channels as a result of the long deactivation time.

Given, that the attenuation of responses arriving at low frequencies in adult neurons is regularly accompanied by a sag [32], the frequency-dependent reduction of the sag should shift or prevent the frequency peak at early stages. Indeed, perinatal cortical neurons steadily attenuated responses arriving at increasing frequencies, a feature provided by non-HCN conductances and by passive membrane properties (Figure 7C/D). In detail, neurons at P0 (n = 7) and at E20 (n = 5) did not have a resonance peak, although each of the neurons had a voltage sag upon the initial hyperpolarization when recorded in ACSF. At the more mature stage of P30, however, each neuron tested had a distinct resonance peak and the average resonance frequency was 2.5 ± 0.3 Hz (n = 9, Figure 7F/G). Phase shift profiles of the example neurons revealed that the neuron at P30 had a positive peak phase followed by a transition to a negative phase compared to only a negative phase in the P0 neuron (see inset Figure 7D/G). Therewith, at perinatal stages the distinctive recognition of inputs based on frequency content, as in later stages [33] is suspended. Our protocol had low frequency components, but our analysis was limited to frequencies above 0.5 Hz to avoid boundary effects [34].

In order to clarify which of the characteristics contributes most to the loss of resonance at P0, we combined certain experimental parameters of *I*_h_ at P0 and P30 from a published model [35]. Only when taking all current characteristics into account (Figure 7E, Table 2), could we reproduce the frequency behavior in perinatal stages (0.7 Hz at P0 (for technical reasons non-measurable *in vitro*) versus 3.5 Hz at P30) accompanied by the P0 specific impedance. In detail, simulated resonance behavior in a model neuron at P0 with a selective replacement of *I*_h_ parameters such as deactivation kinetics or *ĝ*_*h*_ taken from P30 produced a resonance peak at 1.9 Hz or 1.4 Hz, respectively. Additionally, replacement of V_1/2_ or of passive parameters by the respective P30 values had an impact on impedance (Figure 7E). Prolonging the deactivation as in P0 alone in the neuron model at P30 is not sufficient to completely suppress membrane resonance and instead produced a peak at 1.28 Hz (Figure 7H). Phase shift analysis of the model data reflected the experimental results with a pure negative phase in the simulated P0 neurons and a change from positive to negative phase values in the P30 neuron (see insets Figure 7E/H).

**Table 2:** Simulation parameters of the subunit specific model

	***P0 simulation***	***HCN1/4 simulation***		
***Parameter***	**HCN fast**	**HCN slow**	***HCN1***	**HCN4**
*V*_½_ (mV)	−67.1	−65	as HCN fast	−80
*k*	−9.4	−11.8	as HCN fast	−14
*gmt*	0.47	0.506	as HCN fast	0.616
*aNt* (s^-1^)	6.9E^-3^	4.4E^-4^	as HCN fast	8.1E^-4^
z	1.71	2.027	as HCN fast	2.13
*V*_h_ (mV)	−62	−61	as HCN fast	−80
*V*_rev_ (mV)	−36	−36	−25	−25
*ĝ*_h_ (S/cm^2^)	10.6E^-6^	9.4E^-6^	2.2E^-4^	6.9E^-4^
*V*_revleak_ (mV)	−5	5		
*ĝ*_leak_ (S/cm^2^)	1.27E^-5^	6.18E^-4^		
*T* (°C)	32	24		

Taken together, prolonged *I*_h_ deactivation and depolarized *I*_h_ voltage sensitivity led to marked changes in the neuronal frequency response and therewith to a modified sensitivity of the developing neurons against incoming signals.

## Discussion

Our focused systematic approach revealed a hitherto unexpected high number of perinatal *I*_h_-expressing neurons in the rat neocortex. Within a short time period around birth (P0 ± 1 day) *I*_h_ exhibited specific stereotyped changes: *I*_h_ had very low current densities and displayed a high cAMP sensitivity of the half-maximum activation voltage when compared with P30. The most unexpected finding at this age, however, was the very slow current deactivation, which was about 10 times slower than in the adult neurons. Coincidentally the phylogenetically old subunits HCN3 and HCN4 [36] were transiently present in the membrane fraction, HCN1 appeared to be processed differently and HCN2 was absent. The homo- and/or heteromeric interplay of at least two of the present subunits provides a plausible and simple explanation for the concerted *I*_h_ properties at perinatal stages. The properties of *I*_h_ at P0 were remarkably similar, indicating both a tight regulation and a particular function.

### Subunit composition

One of the main functional differences between HCN subunits is their cAMP sensitivity and kinetic behavior [3]. Comparing our functional, mRNA and protein results to known subunit characteristics provides us with possible subunit compositions. At P0 we found a pronounced cAMP sensitivity that excludes monomers or heteromers of HCN3 and HCN1 due to the supposable low cAMP sensitivity in both cases [37]. Therefore, HCN4 is presumably responsible for the cAMP sensitivity, as it is the only cAMP-sensitive subunit we detected in the membrane fraction at P0. Furthermore, activation times of HCN4 homomers alone are too slow [38] to account for the relatively fast current activation. This fast activation could be accounted for by the HCN1 subunit [3]. Although not N-glycosylated at P0, HCN1 could be functionally inserted in the membrane because N-glycosylation is not required for HCN1 surface expression and function in Xenopus oocytes [39]. Pronounced activity leads to the glycosylation of HCN1 and to increased heteromerization with HCN2 in hippocampal neurons [40]. In case such a mechanism would apply for the developing cortex, this could explain the differences in membrane insertion of HCN1 and HCN2 between P0 and P30 despite their similar mRNA expression. Alternatively, if present, HCN3 can act as a negative regulator of HCN2 surface expression [30,41], which would also prevent a faster onset of HCN2 expression. Our data complements the findings that the expression of specific HCN subunits is environmentally regulated and selectively modulated by neuronal activity [29,42,43]. A rapid increase of HCN1 protein levels (30 minutes) can follow NMDA receptor activation [44]. During neocortical development, neuronal activity may initiate the expression of the ‘adult’ HCN1- (and HCN2-) driven *I*_h_, because patterns of distribution of HCN1 channels depend on network activity [12,45]. However, as HCN2 surface expression is enhanced by phosphorylation [46] and phosphorylation sites for HCN1 exist [47], we cannot exclude a fast alteration of membrane protein surface expression by phosphorylation [48].

Expression studies have shown that both homo- and heteromeric HCN tetrameres are functional [30,49-51]. We argue from our data that the membrane of the perinatal cortical plate neurons must contain at least two distinct HCN subunits with different cAMP sensitivity and kinetics. In line, our HCN1 and HCN4 co-expression experiments, that revealed *I*_h_ characteristics similar to our results in P0 neurons, serve as a proof of concept. The only difference we found was the cAMP dependence of the current activation in the co-expression experiments, which we did not observe similarly pronounced in neocortical neurons. This might be due to a strict regulation of expression and trafficking in the neuron versus a less defined situation in the heterologous system, the different cellular context or a different degree of heteromerization. Taken together, our data suggest that either HCN subunits differentially heteromerize or different homomeric HCN channels co-localize within the same cell [30,43] at P0. We propose that the coordinated neuronal HCN expression is influenced by neuronal activity as part of a developmental program.

### Alternative explanations

Although it is tempting to link HCN protein expression patterns to functional alterations, such a link does not rule out an impact of other factors on *I*_h_ during development. For instance, an interaction of HCN subunits with a number of proteins is known [3]. One of these proteins is TRIP8b that can act on trafficking and gating and interacts at least with HCN1, furthermore, several TRIP8b isoforms exist that influence current density to a different extent [52]. In the hippocampus the expression of specific TRIP8b isoforms is developmentally regulated [53], hence a similar regulation of TRIP8b is imaginable in the developing neocortex. Also, the fast modulation of tyrosine phosphorylation by Scr kinase in the C-linker region [54] may play a role in channel activation and gating especially in light of the influence of growth factors on protein kinases [55]. As disruption of lipid rafts caused an about 10 mV depolarization of the voltage sensitivity and a twofold increase in the time constant of deactivation [56], HCN channels may not be properly localized to lipid rafts perinatally. Finally, a conformational stabilization of an open state, as it can be induced by Cd^2+^ binding [57], seems possible, because the current behavior reminds of a ‘locked open’ state. All these examples merely discuss established modulations with the potential to explain the unexpected behavior of HCN channels at P0, but do not exclude others.

### Technical considerations

Whereas whole-cell patch-clamp is a convenient method, it has limitations with putative relevance to our approach: first, shunting conductances, second, space-clamp issues and third, exchange of cytoplasmic components with pi-pette contents. First: as in our study, the small neurons at early time-points had input resistances above 1 GΩ a shunt between pipette and cell membrane must be considered. Such a shunt could explain the measurement of depolarized membrane potentials [58]. Aware of this, we aimed to keep the ratio of seal versus input resistance as high as possible. This was reflected by relatively hyperpolarized membrane potentials in ACSF and the adjacency of membrane potential and *I*_h_ reversal potential after pharmacological isolation of *I*_h_. Moreover, the shunt did not appear to impair voltage control, because we saw a slightly depolarized voltage-dependence instead of the expected hyperpolarized voltage-dependence in case of poor voltage control of *I*_h_ activation and a pronounced negative shift without cAMP. Second: although the fast activation kinetics at P0 might reflect a positive shift in voltage relation, the marginal increase of *I*_h_ activation kinetics at P30 might arise from space-clamp issues [25] due to the isochronal developmental increase in morphological complexity. Moreover, space-clamp issues cannot explain the different developmental time course of *I*_h_ activation and deactivation. The high input resistance of the neurons at P0 also argues against electrotonic coupling under the conditions of our study, which is in line with earlier studies [59]. Third: the morphological compactness of P0 neurons favors a faster solution exchange between the pipette and intracellular compartments compared to morphologically more complex neurons. In order to exclude such diffusional differences, we recorded *I*_h_ with a delay of >10 minutes after establishing the whole cell mode.

### Functional relevance

The transient HCN subunit expression of HCN3 and HCN4 may indicate a requirement of these subunits during specific stages of neuronal development. This is supported by the importance of HCN4 for early embryonic heart development [60]. In addition, the dynamic HCN1, HCN2 and HCN4 regulation is reminiscent of earlier results on hippocampal development [6,43,61] and partially on thalamic development [62]. Hence, considering our data, this may point to a more widespread neuronal mechanism. Moreover, the finding of strong HCN3 mRNA expression in the human fetal brain compared to HCN1 [37] might suggest a general developmental rather than a species-specific effect.

Given the multiple functional implications of *I*_h_[3], the putative impact of *I*_h_ maturation on neuronal development is manifold [63]. *I*_h_ mediates a main developmental change in postnatal layer 5 pyramidal neurons [17], the electrotonic separation of neuronal compartments. *I*_h_-increase contributes to the dramatic decrease in input resistance [17], regulates the responsiveness to small inputs [64] and neuronal excitability. Due to its complex influence on dynamic synaptic events, *I*_h_ enables a balance of excitation and inhibition in cortical networks and may enhance the establishment and stabilization of neuronal circuits [6,17]. *I*_h_ also interplays with ‘passive’ membrane properties to determine resonance behavior at and below resting potential, which results in an impedance peak at 2.5 Hz in mature layer 5 pyramidal neurons (this study, [32]). Nevertheless, at P0 *I*_h_ failed to generate a detectable resonance peak *in vitro* as well as *in silico*. Whether this is a specific neuronal reaction to the frequency content of the incoming signals and as such an adaptation to rarely arriving inputs, or it simply represents a side effect of the developmental-driven changes in HCN composition needs to be finally resolved.

Although we did not investigate network activity directly in our study, the observed early *I*_h_ properties appear to be temporally related to the occurrence of spontaneous, and widespread synchronous activity [65] emerging in the neocortex just before birth, peaking at P0, and ceasing by about P5 [7]. Therefore, a switch in channel subunits might reflect a functional shift from slow rhythmic events to non-rhythmic events that is accompanied by an input resistance drop [66]. The early appearance of HCN4- and/or HCN3-mediated slow deactivation kinetics may partially underlie the slow rhythmicity at P0, given that prolonged activation of *I*_h_ is associated with rhythmic network activities [67,68]. Therewith, the role of *I*_h_ phenotype in the neocortex in particular seems to go beyond the adaptive role suggested for the hippocampus [61].

## Conclusion

For understanding neocortical development in relation to electrical activity, it is important to unravel conductances and their properties during early developmental time points. For this reason, we studied the perinatal occurrence of *I*_h_ in the developing neocortex and identified novel aspects of *I*_h_ characteristics in cortical plate neurons. Our data indicate a rapid increase of *I*_h_ right after birth and show that *I*_h_ appears earlier than described for phylogenetically older cortical areas, as for instance the hippocampus. Cyclic AMP sensitivity is increased and the deactivation is slowed in a narrow perinatal time frame when compared to adulthood, pointing to a high impact of changes in cAMP levels. The combination of these current properties causes either a lack of neuronal membrane resonance or a shift to very low frequencies, which makes them putatively relevant for cortical network activity. The distinct functional features are related to the membrane appearance of HCN channels composed of phylogenetically old HCN subunits with slow kinetics. Taken together, these findings hint at a so far undescribed mechanism of a neocortical developmental change in HCN processing.

## Methods

### Animals

All procedures in this study were performed in accordance with the European Communities Council Directive of 24 November 1986 (86/609/EEC). Approval of experiments was obtained from the local ethics bodies of Mecklenburg-Vorpommern and Berlin (LAGeSO: T0108/11 and T0181/11). For experiments Wistar rats of both sexes were used (Charles River or from the Charité central animal facility: FEM). Timed pregnant Wistar rats were maintained in our respective local animal facility from embryonic day 9 (E9) to 18 (E18). The day of birth was designated as P0. Animals were kept at a 12-hour light/dark cycle with food and water *ad libitum*.

### Slice preparation and patch-clamp recordings

Rats were deeply anesthetized with isoflurane or ether before decapitation. Brains were quickly removed and placed in 4°C ACSF comprised of (in mM): 117 NaCl, 3.5 KCl, 1.25 NaH_2_PO_4_, 26 NaHCO_3_, 2 CaCl_2_, 2 MgCl_2_/MgSO_4_ and 10 glucose (all from Sigma-Aldrich, St Louis, MO, USA). Acute coronal brain slices of 400 μm thickness were cut and allowed to recover for 30 to 40 minutes at 34 ± 1°C before being stored at room temperature for the rest of an experimental day. Individual slices were transferred to a submerged recording chamber (1 to 2 ml/min ACSF at 32°C) and neurons were visualized using infrared differential interference contrast video microscopy with a 40- or 60-fold magnification water-immersion objective (Axioskop 2 FS, Carl Zeiss AG, Oberkochen, Germany).

Whole-cell current and voltage recordings were performed in layer 5 neurons when layers were clearly distinguishable. The pipette solution contained (in mM): 120 KMeSO_4_ (ICN Biomedicals, Eschwege, Germany), 20 KCl, 14 Na-phospocreatine, 0.5 EGTA, 4 NaCl, 10 HEPES, 4 Mg^2+^**-**ATP and 0.3 Tris-GTP with or without 0.1 cAMP (all from Sigma-Aldrich), (pH 7.25, 288 mOsm). Pipettes filled with that solution had a tip resistance between 3 and 5 MΩ.

For subsequent pharmacological isolation of *I*_h_ we modified the bath ACSF (with 10 mM K^+^, MgSO_4_ replaced by MgCl_2_ and NaH_2_PO_4_ omitted, all from Merck). Additionally, we blocked relevant confounding currents by 200 μM Ba^2+^ (Merck KGaA, Darmstadt, Germany), 1 μM tetrodotoxin (TTX) (Tocris, Bristol, UK), 1 mM Ni^2+^, 20 μM 6-cyano-7-nitroquinoxaline-2,3-dione (CNQX), 25 μM D-(−)-2-amino-5-phosphonopentanoic acid (DAP-5), 10 μM bicuculline, 5 mM 4-AP and 10 mM tetraethylammonium (TEA) (all from Sigma-Aldrich). In some experiments we blocked phospholipase C by adding U73122 (1 or 10 μM, Sigma-Aldrich, [69]) intracellularly. The identity of the current was confirmed at all ages by blocking the remaining current with 4-ethylphenylamino-1,2-dimethyl-6-methylaminopyrimidinium chloride (ZD7288, Tocris) or cesium (Cs^+^, Sigma-Aldrich).

Data from patch-clamp recordings were collected with an EPC-10 double amplifier (HEKA, Lambrecht, Germany), digitized (minimum of 10 kHz, after Bessel filtering at 2.5 kHz) and stored using Pulse or PatchMaster software (HEKA).

The electrical resonance of neurons was recorded in ACSF (KCl changed to 2.5 mM and NaCl adjusted accordingly) at −64 mV for P0 and at −69 mV for P30. The resonance was estimated with the impedance (Z) amplitude profile (ZAP) method [33] with an in-house program written in NI LabVIEW 8.5 (National Instruments Inc., Austin, TX, USA). A sinusoidal current with constant amplitude and linearly increasing frequency (0 to 10 Hz at P30 and 0 to 20 Hz at P0) was injected. To maintain a linear current–voltage relationship the amplitude of the ZAP current was adjusted to a maximum voltage deflection (peak to peak) below 25 mV. Recordings were sampled at 1 kHz after low pass filtering with a cutoff frequency of 400 Hz. The frequency profile of the impedance was achieved by dividing the magnitudes of Fast Fourier transformed voltage and current traces. Phase plots were obtained by subtracting corresponding phase angles. The impedance plot was smoothed with a moving average and the impedance peak was determined by a least squares algorithm.

Liquid junction potentials (approximately 10 mV) were not corrected for. Series resistance (R_s_) was monitored regularly during an experiment and no change was observed between ages (*P* = 0.3). Input resistance was calculated from steady-state voltages after current injections around membrane threshold with Ohm’s law. Whole cell capacity (C) was estimated from three responses to square voltage injections (ΔV = 10 mV) by use of the equation C = Q/ΔV, where Q represents the calculated integral of the current response. Normalized tail current amplitudes were plotted against the preceding step voltage (V) and fitted with the Boltzmann equation:

$$I\left(V\right)=\left(a_{1}-a_{2}\right)/\left(1+e^{((V-V}{{}_{1/2}}^{)/k)}\right)+a_{2}$$

V_1/2_, the midpoint of voltage activation, and k, the slope of the curve, were identified to determine the voltage sensitivity of the *h*-channel. Kinetic analysis was performed on non-subtracted traces. Best descriptions of the *I*_h_ activation were achieved by double-exponential fits with first- (τ_fast_) and second-order (τ_slow_) time components. The current deactivation was best fitted by a single exponential equation. The amplitude of *I*_h_ was obtained by measuring the steady-state current and subsequently subtracting the current after ZD7288 block and/or leak subtraction protocols with similar results [70]. The reversal potential was determined by stepping to different test potentials after fully activating the *I*_h_ (step potential −130 mV for 2 seconds) and linear regression of tail current peak amplitudes (measured as described for voltage sensitivity). Analysis and exponential fits of the current and voltage traces were performed with PulseFit or FitMaster (HEKA) and Origin 7.x (OriginLab Corp., Northampton, MA, USA).

### Cell culture and electrophysiology of HEK cells

HEK293 cells were routinely maintained at 37°C with 95% O_2_ and 5% CO_2_ in Dulbecco’s Modified Eagle Medium supplemented with 10% calf serum, 200 mM L-glutamine and 100 U/ml penicillin/streptomycin (all PAN-Biotech GmbH, Aidenbach, Germany). For electrophysiological experiments, we plated HEK cells at low density on poly-L-lysin-coated coverslips and co-transfected them the next day by calcium phosphate precipitation with cloned rHCN1 [31] and hHCN4 (a gift from Juliane Stieber, University of Erlangen) at a 1:1 ratio. Over-expressing cells were placed on the stage of a Zeiss Axiovert S100 (Carl Zeiss AG, Oberkochen, Germany) identified by fluorescence and visualized with phase contrast. Extracellular solution was gravity applied and had the following composition (in mM): 120 NaCl, 10 KCl, 1.8 CaCl_2_, 0.5 MgCl_2_, 10 HEPES, 10 glucose, 10 TEA, and 5 4-aminopyridine, pH 7.4. Experiments were carried out at room temperature (22 to 24°C). Recordings and data analysis were performed as described for the slice experiments.

### Computational models

Our models were implemented in Scilab 5.0.1 (Scilab Consortium, INRIA, ENPC and contributors, 1989 to 2008) with simulation control programmed in LabVIEW 8.5 (National Instruments Inc.). We based the resonance simulation on an established model that was derived from the interplay of *I*_h_ and passive cell properties [35]. The parameters were set to the experimental observations *in vitro* that were for P0: *V*_*½*_ = −65.6 mV*; k* = 7.8*; ĝ*_*h*_ = 6.95E^-10^ S; *V*_rev_ = −40.9 mV; *time constant* (*τ*) = 1219.4 ms; *capacitance (C)* = 46.43 pF; *ĝ*_*leak*_ *=* 3.04E^-10^ S and for P30: *V*_*½*_ = −80.8 mV; *k* = 12.7; *ĝ*_*h*_ *=* 8.74E^-9^ S; *V*_rev_ *= −*37 mV; *τ* = 153.4 ms; *C* = 135 pF; *ĝ*_*leak*_ = 7.16 E-9 S. The conductivity parameters were scaled according to the cell capacitance (*C*_P0_/*C*_P30_ = *ĝ*_P0_/*ĝ*_P30_). For the simulation run the membrane potential was set to −70 mV.

Our second - subunit specific - model simulated the net effect of the expression of kinetically different subunits. The simulation was performed for two instances with two independent first-order kinetic models. Both, the fast and the slow current component, were described by

$$I_{\text{hfast},\text{slow}}={\hat{g}}_{h}*\text{ l}\left(V_{m},t\right)*\left(V_{m}–V_{\text{rev}}\right),$$

where *ĝ*_h_ was the maximum HCN channel conductance, *V*_*m*_ the membrane potential at time *t* and *V*_rev_ the reversal potential of the HCN channel. The proportion of open HCN channels described by this equation evolved according to a first order kinetic:

$$\frac{dl}{dt}=\frac{\left(l\left(V_{m}\right)-l\right)}{t\left(V_{m}\right)}$$

Here the steady-state activation is calculated with

$$l\left(V_{m}\right)=\frac{1}{1+e^{\frac{-(V)m-V_{1/2}}{k}}}$$

and the kinetics with

$$\tau \left(V_{m}\right)=\frac{b}{q_{T}*aNt*\left(1+a\right)}$$

where *a* = e^0.0378 * *z* * (*Vm* – *Vth*)^ and *b* = e^0.0378 * *gmt* * *z* * (*Vm* – *Vth*)^. The temperature dependence of the channel kinetics was described by *q*_T_ = *q*_10_^(*T* – 33)/10^, where *T* was the nominal temperature in Celsius and *q*_10_ set to 4.5 [71]. The complete *I*_h_ (*I*_htot_) was calculated by summation of the two *I*_h_ subunits *I*_htot_ = *I*_hfast_ + *I*_hslow_. The parameters describing *I*_hfast_ and *I*_hslow_ were allowed to vary freely with the only constraint being *V*_revfast_ = *V*_revslow_. To fit the *I*_htot_ trace to the experimentally recorded *I*_h_ traces, it was necessary to add a voltage-independent leak current to the simulated *I*_htot_. This leak current was described by

$$I_{\text{leak}}={\hat{g}}_{\text{leak}}*\left(V_{m}-V_{\text{revleak}}\right),$$

where *ĝ*_leak_ was the conductance of the leak channel and *V*_revleak_ the corresponding reversal potential.

We used this model implementation for two different approaches. In the first model (‘P0 simulation’, see Table 2) we aimed to reproduce the fast activation/slow deactivation found in P0 cells *in vitro* with two kinetically different subunits. Here, the parameters describing the voltage dependence (*V*_½_ and *k*) and kinetics (*gmt*, *aNT*, *z*, and *V*_th_) were set according to our previous rHCN1 measurements in HEK cells [31]. As a simplifying assumption, the slow subunit compromises the decelerating effects of the putatively contributing other subunits HCN2, HCN3 and HCN4 and its parameters were allowed to vary freely in order to fit the original P0 *in vitro* trace.

The second model (‘HCN1 and HCN4 simulation’, see Table 2) mirrors the HCN1/HCN4 co-expression experiments. By varying the proportions of HCN1 and HCN4 and keeping the sum of both subunits constant we obtained different *I*_h_ kinetics (compare Figure 6G).

### Real-time PCR

Neocortical tissue was immediately snap-frozen following its collection from three sets of six animals of each age. Total RNA was isolated according to the TRIzol protocol (Life Technologies Corp., Carlsbad, CA, USA). cDNA was synthesized using the High-Capacity cDNA Archive Kit (Applied Biosystems, Foster City, CA, USA) according to the manufacturer's protocol. For qRT-PCR, TaqMan Universal PCR Master Mix Kit (Applied Biosystems) was used. Gene expression assays for HCN1 (Rn00670384_m1), HCN2 (Rn01408572_mH), HCN3 (Rn00586666_m1), or HCN4 (Rn00572232_m1) were used. Glyceraldehyde-3-phosphate dehydrogenase (GAPDH) (Rn99999916_s1) and hypoxanthine guanine phosphoribosyl transferase (HPRT) (Rn01527838_g1) were used as internal controls. Reactions were run on ABI PRISM™ 7700 Sequence Detection System (Applied Biosystems). Standard curves were produced with serial dilutions of cDNA from rat neocortex P5 with amplification efficiency between 95% and 100%. To determine the relative gene expression in each experiment samples were double-tested and a no template control included. Each result is the average of three separate experiments.

For quantitative comparison of HCN gene expression, data from each qRT-PCR run were analyzed using the 7500 Fast System software (Applied Biosystems). The value of the noise fluorescence, usually indicated as the base line of the run, was automatically determined. The threshold value was manually set to 0.05. The threshold cycle (C_T_) was automatically calculated and used to quantify the starting copy number of the target mRNA. Expression of HCN genes in neocortical tissue was normalized to GAPDH and HPRT expression by means of the 2-ΔΔC_T_ method [72].

### Membrane and cytosolic protein preparation and immunoblotting

Anesthetized animals (P0 and P30) were decapitated. Neocortical tissue was rapidly dissected and immediately frozen in liquid nitrogen or dry ice. Tissue homogenization was performed in lysis buffer containing 20 mM Tris–HCl (pH 7.5), 0.25 M sucrose, 1 mM EGTA, and 5 mM EDTA which was supplemented with a protease inhibitor cocktail (PIC Complete, diluted according to the manufacturer; Roche Applied Science, Mannheim, Germany) and a phosphatase inhibitor cocktail (PhosSTOP, diluted according to the manufacturer; Roche). As positive controls we used protein extracts from HEK293 cells over-expressing either pIRES2-dsRed-rHCN1 [31], pIRES2-eGFP-rHCN2 (kindly provided by Shigetada Nakanishi), rHCN3-pcDNA1 (kindly provided by Prof. Müller, Research Centre Jülich), or hHCN4-pcDNA3 (kindly provided by Juliane Stieber, University of Erlangen). For protein extraction HEK293 cells were plated at a density of 20,000 cells/cm² (cultured as described above) and transfected (calcium phosphate) the next day with the constructs. Two days after transfection HEK293 cells were washed with PBS and harvested with a cell scraper in lysis buffer.

Samples, tissue or cells, were sonicated three times for 3 seconds followed by a 30-minute incubation on ice and a centrifugation at 150,000 *g* for 20 minutes at 4°C. The supernatant containing cytosolic proteins was stored at −20°C. The pellet containing membrane fractions was resuspended in lysis buffer with 1% Triton X-100 followed by incubation on ice for 30 minutes and a subsequent centrifugation at 2300 *g* for 10 minutes at 4°C. The resulting supernatant containing membrane proteins was collected. For all samples protein concentration was determined using the BCA method.

For detection of potential N-glycosylation, P0 and P30 rat neocortical membrane protein lysates were treated with peptide-N-glycosidase F (PNGase F, Roche) as described by [73]. Controls of rat neocortex protein lysates were treated using the same process, but without PNGase F.

Membrane and cytosolic samples were subjected to 10% SDS-PAGE under reducing conditions and then blotted to a nitrocellulose membrane (Whatman, Dassel, Germany) by semidry protein transfer. Blots were blocked for one hour at room temperature in 10% non-fat powdered milk diluted in 1 × PBS. We used antisera against HCN1 (Alomone Labs Ltd., Jerusalem, Israel), HCN2 (Alomone Labs), HCN3 (Alpha Diagnostic, San Antonio, TX, USA), and HCN4 (Alomone Labs). Beta-actin (Sigma-Aldrich, Germany) expression was used as an internal control. After three washes in 1 x PBS, the membranes were incubated with a 1:5000 dilution of an anti-mouse or anti-rabbit horseradish peroxidase-labeled antibody (GE Healthcare Europe GmbH, Freiburg, Germany) for two hours at room temperature. Immunoreactive bands were detected using ECL western blotting detection reagents (GE Healthcare).

Antibody specificity and exclusion of cross-reaction between the HCN subunits was confirmed by immunoblots with membrane and cytosolic fractions of HCN1-HCN4 transfected HEK293 cells. In control western blots all used HCN antibodies showed specificity to their target proteins, but no cross-reactivity with other members and recognized the protein in the membrane protein fraction at the expected size (see Additional file 1).

### Statistics

Statistical analysis was carried out with SPSS (10.0, IBM Corp., Somers, NY, USA), StatView (4.57 Abacus Concept Inc., Berkeley, CA, USA) or Origin (7.×, OriginLab Corp.). For comparisons between two groups we used a Mann–Whitney *U* test when n < 8. Multiple group comparisons were performed with a one-way ANOVA combined with a post hoc Tukey test. All tests were performed as indicated. All data are presented as mean ± standard error of the mean (SEM). In case box-plots were used, a line comprising the box and individual data points indicates the mean. The minimum and maximum of boxes indicate the 25^th^ and 75^th^ percentile.

## Abbreviations

ACSF: artificial cerebrospinal fluid; EGTA: ethylene glycol tetraacetic acid; GAPDH: glyceraldehyde-3-phosphate dehydrogenase; HCN: hyperpolarization-activated cyclic nucleotide-gated; HPRT: hypoxanthine guanine phosphoribosyl transferase; PBS: phosphate-buffered saline; PNGase F: peptide:N-glycosidase F; PIP_2_: phosphatidylinositol 4,5-bisphosphate; PLC: phospholipase C; qRT-PCR: quantitative real-time polymerase chain reaction.

## Competing interests

The authors declare that they have no competing interests.

## Authors’ contributions

AB, NR and US designed and performed the *in vitro* experiments, KS and US designed and KS performed the *in silico* experiments, and AUB and US designed the molecular biology experiments. Data was analyzed and interpreted by all authors. The article was drafted by US and written by US and AB with substantial input from KS and AUB and critical comments from NR. All authors approved the final version of the manuscript.

## Supplementary Material

Additional file 1 ** Control western blots for HCN1-4 antibodies.** Control western blots of lysates from HEK cells over-expressing HCN1-HCN4. The western blots show the specificity of the used HCN antibodies to their respective target proteins. Each antibody (HCN1-HCN4) recognized the respective over-expressed protein in the membrane protein fraction at the expected size and showed no cross-reactivity with other family members. As loading controls the western blots were probed with beta-actin. Click here for file
