# Supplementary material for: Distinct perinatal features of the hyperpolarization-activated non-selective cation current Ih in the rat cortical plate
Source: Neural Dev. 2012 Jun 13;7:21. doi: 10.1186/1749-8104-7-21 (PMC3518177; doi:10.1186/1749-8104-7-21)

Distinct Perinatal Features of the Hyperpolarization-Activated Non-selective Cation Current I_h in the Rat Cortical Plate

Arne Battefeld, Nino Rocha, Konstantin Stadler, Anja U. Bräuer and Ulf Straus

Additional file 1

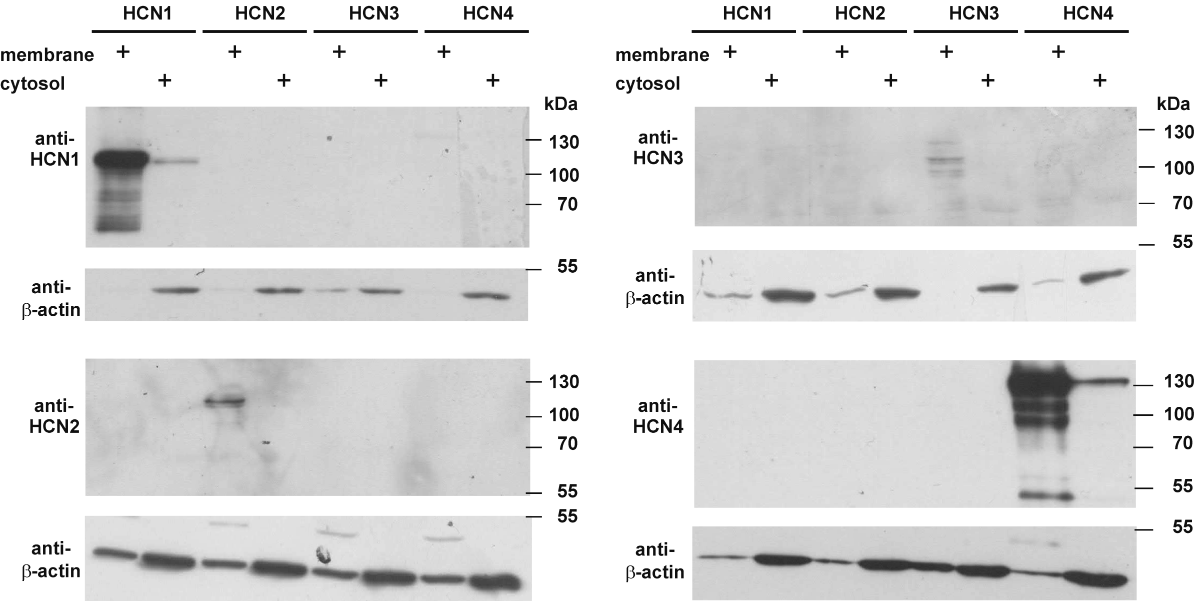

Supplement: Additional file 1 — Control western blots for HCN1-4 antibodies. Control western blots of lysates from HEK cells over-expressing HCN1-HCN4. The western blots show the specificity of the used HCN antibodies to their respective target proteins. Each antibody (HCN1-HCN4) recognized the respective over-expressed protein in the membrane protein fraction at the expected size and showed no cross-reactivity with other family members. As loading controls the western blots were probed with beta-actin. [file 1749-8104-7-21-S1.pdf]
